# Supplementary material for: An In Vitro and In Vivo Assessment of Antitumor Activity of Extracts Derived from Three Well-Known Plant Species
Source: Plants (Basel). 2023 Apr 29;12(9):1840. doi: 10.3390/plants12091840 (PMC10180766; doi:10.3390/plants12091840)
Supplement: Supplementary file 1 [file plants-12-01840-s001.zip › plants-2349954-supplementary.pdf]

An In Vitro and In Vivo Assessment of Antitumor Activity of Extracts Derived from Three Well-Known Plant Species

Octavia Gligor [1], Simona Clichici [2], Remus Moldovan [2], Nicoleta Decea [2], Ana-Maria Vlase [1], Ionel Fizeșan [3,*], Anca Pop [3], Piroska Virag [4], Gabriela Adriana Filip [2,*], Laurian Vlase [5] and Gianina Crișan [1]

1 Department of Pharmaceutical Botany, Iuliu Hatieganu University of Medicine and Pharmacy, 8 Victor Babes Street, 400347 Cluj-Napoca, Romania
2 Department of Physiology, Iuliu Hatieganu University of Medicine and Pharmacy, 8 Victor Babes Street, 400347 Cluj-Napoca, Romania
3 Department of Toxicology, Faculty of Pharmacy, Iuliu Hatieganu University of Medicine and Pharmacy, 8 Victor Babes400347 Cluj-Napoca, Romania
4 Department of Radiobiology and Tumor Biology, Oncology Institute "Prof. Dr. Ion Chiricuță", 34-36 Republicii Street, 400015 Cluj-Napoca, Romania
5 Department of Pharmaceutical Technology and Biopharmaceutics, University of Medicine and Pharmacy, 8 Victor Babes Street, 400347 Cluj-Napoca, Romania
* Correspondence: ionel.fizesan@umfcluj.ro (I.F.); gabriela.filip@umfcluj.ro (G.A.F.)

4. Materials and Methods

4.3. HPLC-MS Analysis of the Plant Extracts

The HPLC system used was an 1100 series Agilent Technologies model (Darmstadt, Germany) consisting of a G1312A binary pump, an in-line G1379A degasser, an G1329A autosampler, a G1316A column thermostat and an Agilent Ion Trap Detector 1100 SL, and a UV detector.

Chromatographic separation was performed on a Zorbax SB-C18 (100 mm x 3.0 mm i.d., 3.5 µm) column (Agilent Technologies) equipped with a Zorbax SB-C18 precolumn with a mixture of methanol / 0.1% (v/v) acetic acid in water 52/48 (v/v), at 50 ºC with a flow rate of 1 mL/min. The sample injection volume was 1 µL. The detection of analytes was performed in MS/MS mode, positive ionization, using an ion trap mass spectrometer equipped with an atmospheric pressure chemical ionization (APCI) ion source: capillary 4000 V, heater 400ºC, nebulizer 60 psi (nitrogen), dry gas nitrogen at 7 L/min, dry gas temperature 300ºC. In these chromatographic conditions, the retention times were 1.4 min for 8-epi-xanthatin and 1.7 min for xanthatin, respectively. The mass spectrometry transitions used for quantification of analytes were m/z 247 > m/z (229.2; 183.2; 187.2; 201.2) for both compounds (as they are isomers). The calibration curves were linear for both analytes, in range of 1-20 µg/mL [105].

For the separation of the isoflavones formononetin and ononin, a Zorbax SB-C18 (100 µm x 3.0 µm i.d., 3.5 µm particle size) reverse-phase analytical column was used. The temperature was set to 48 °C. The separation of the two compounds was carried out using a mobile phase containing a mixture of acid 0.1% (v/v) in water (phase A) and methanol (phase B). The elution was in gradient mode, i.e., 20% phase B for the first 2 min, at 10 min 40% phase B, at 10.5 min 40% phase B, at 11.5 min 45% phase B. This continued up until 17 min. The flow rate was set to

1 mL/min. For the detection and quantification of the two isoflavones, the HPLC system was linked with an Agilent 1100 Ion Trap SL mass spectrometer and operated with an electrospray (ESI) ion source in negative ion mode. Nitrogen at 65 psi was used for vaporization, as well as dry gas, with a 12 L/min flow rate, temperature being set to 360 °C. Capillary potential +2500 V. The calibration curves for the two isoflavones were in the range of 40-4000 ng/ml. A quadratic model and 1/y weighting scheme were used for the fitting of the calibration curves. The accuracy of the calibration points, for both formononetin and ononin, was of a maximum of ±15%.

For the isoflavone biochanin A, the chromatographic separation was performed with Zorbax SB-C18 (100 mm x 3.0 mm i.d., 3.5 µm) column (Agilent Technologies) equipped with a Zorbax SB-C18 precolumn with a mixture of methanol / 0.1% (v/v) acetic acid in water 43/57 (v/v), at 48 ºC with a flow rate of 1 mL/min. The sample injection volume was 1 µL. The detection of the analytes was performed in MS/MS mode, negative ionization, using an ion trap mass spectrometer equipped with an electrospray ionization (ESI) ion source: capillary 3500 V, nebulizer 60 psi (nitrogen), dry gas nitrogen at 12 L/min, dry gas temperature 300ºC. In these chromatographic conditions, the retention time of analyte was 2.6 min. The mass spectrometry transition used for quantification of analyte was m/z 283 > m/z 268. The calibration curves were linear for all three analytes in the range of 0.25-10 µg/mL [105-109].

For the diCQA compounds, the chromatographic separation was also performed on a Zorbax SB-C18 (100 mm x 3.0 mm i.d., 3.5 µm) column (Agilent Technologies), but equipped with a Zorbax SB-C18 precolumn with a mixture of methanol / 1mM ammonium acetate in water 14/86 (v/v). The temperature was also 48 °C with the flow rate of 1 mL/min. The sample injection volume was 5 µl. The detection of the diCQA compounds was performed in MS/MS mode, negative ionization, in the following conditions: capillary 3500 V, nebulizer 60 psi (nitrogen), dry gas nitrogen at 12 L/min, dry gas temperature 350 ºC. Thus, retention times were 4.2 min for 3,5-diCQA, 5.7 min for 4,5-diCQA and 6.5 min for 3,4-diCQA, respectively. The mass spectrometry transitions used for quantification of analytes were m/z 515 > m/z 353 for all three compounds (as they are isomers). The calibration curves were linear for all three analytes in range of 1-15 µg/mL. For the 4-O-CQA, a reverse-phase analytical column was used (Zorbax SB-C18, 100 × 3.0 µm i.d., 3.5 µm particle size). The injection volume was 5 µL and the flow rate was 1 mL/min. The UV and MS modes were used for this detection. The UV detector wavelength was set at 330 nm up until 17.5 min in the analysis, to be reset to 370 nm for the remainder of the analysis. The source of ionization for this determination was an electrospray operating in negative mode. The MS system operated in monitoring specific ions mode. Separation was done using a mobile phase of methanol:acetic acid 0.1% (v/v). The elution took place in a binary gradient. A linear gradient was maintained in the beginning, i.e., from 5% to 42% methanol at 35 min, and further changed to an isocratic elution for the ensuing 3 min (with 42% methanol). Finally, the column was rebalanced using 3% methanol [105,108,109].

For the two diterpenoid compounds, cafestol and kahweol, the chromatographic separation was performed on a Zorbax SB-C18 (100 mm x 3.0 mm i.d., 3.5 µm) column (Agilent Technologies) equipped with a Zorbax SB-C18 precolumn with a mixture of methanol/acetic acid

0.1% (v/v) in water 65/35 (v/v), at 50 ºC with a flow rate of 1 mL/min. The sample injection volume was 2 µl. The detection of analytes was performed in MS/MS mode, positive ionization, using an ion trap mass spectrometer equipped with an APCI ion source: capillary 3000 V, heater 450ºC, nebulizer 60 psi (nitrogen), dry gas nitrogen at 7 L/min, dry gas temperature 350ºC. In these chromatographic conditions, the retention times were 4.3 min for KA and 4.8 min for cafestol, respectively. The mass spectrometry transitions used for quantification of analytes were m/z 297.2 > m/z 279.2 for kahweol and m/z 299.2 > m/z (253.2, 281.2) for cafestol, respectively. The calibration curve was linear for both analytes, in range of 2-15 µg/mL.

Data Analysis (v5.3) and ChemStation (vA09.03) software from Agilent Inc. (Santa Clara, CA, USA) were employed to acquire and analyze the obtained chromatographic data.

Examples of the respective resulted chromatograms can be found in Figures S1-S4.

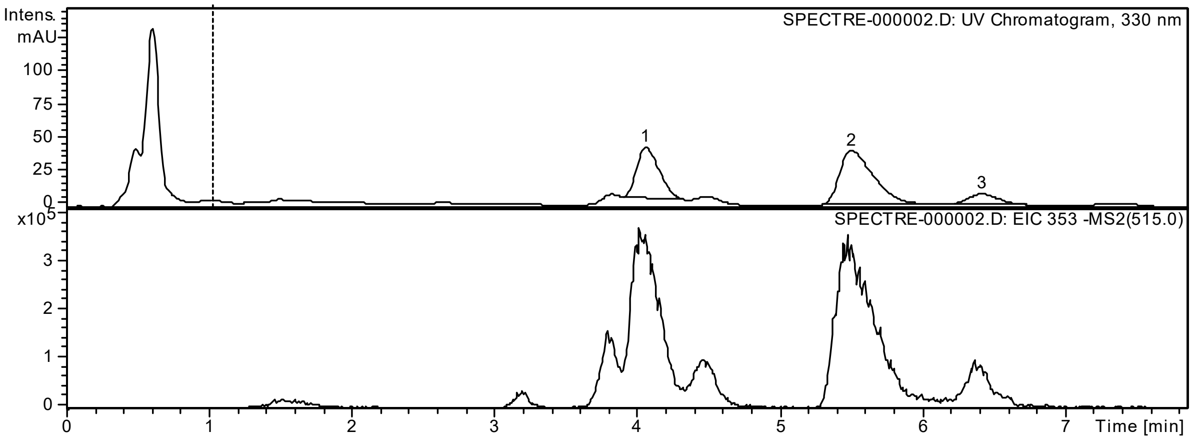

Figure S1. Chromatogram of *Xanthium spinosum* L. S60 extract: upper spectra: UV chromatogram; lower spectra: MS extracted ion chromatogram: (1) 3,5-diCQA, (2) 4,5-diCQA, (3) 3,4-diCQA.

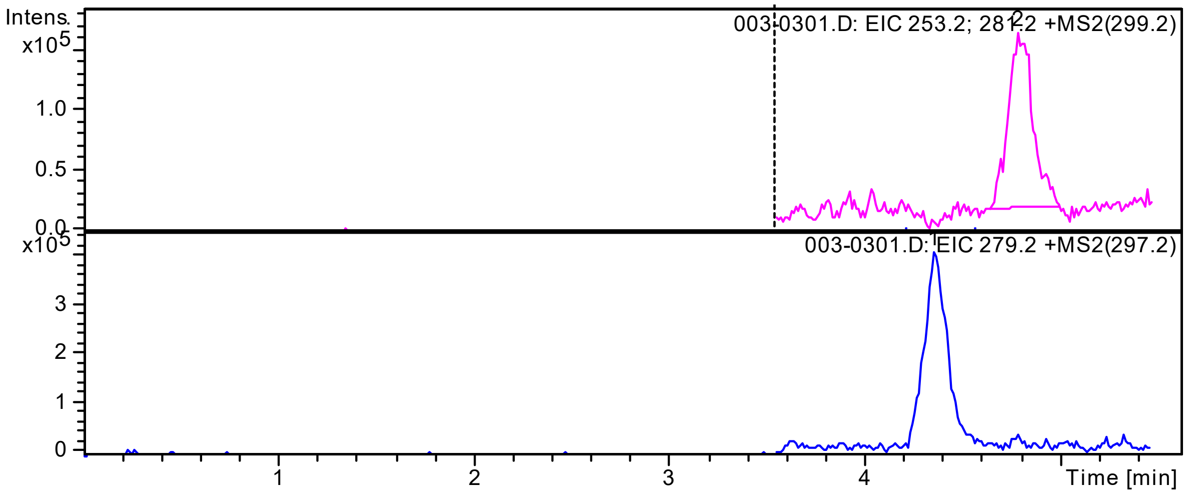

Figure S2. MS chromatogram of green coffee beans S60 extract: upper spectra – cafestol extracted ion chromatogram; lower spectra – kahweol extracted ion chromatogram.

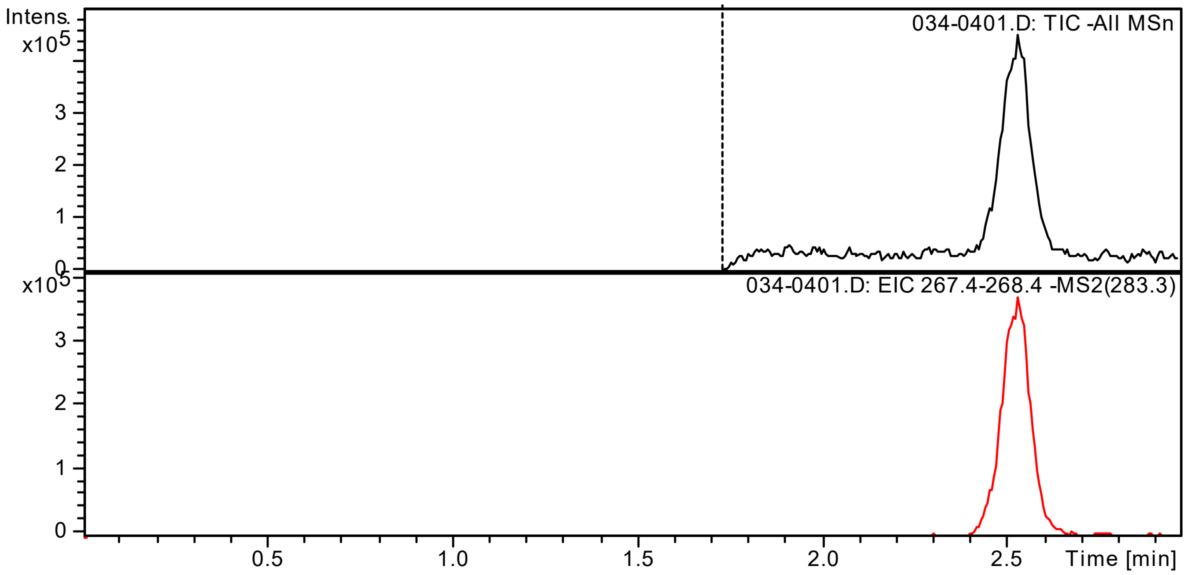

Figure S3. MS chromatogram of *Trifolium pratense* L. S60 extract: upper spectra: total ion chromatogram; lower spectra: extracted ion chromatogram for Biochanin A.

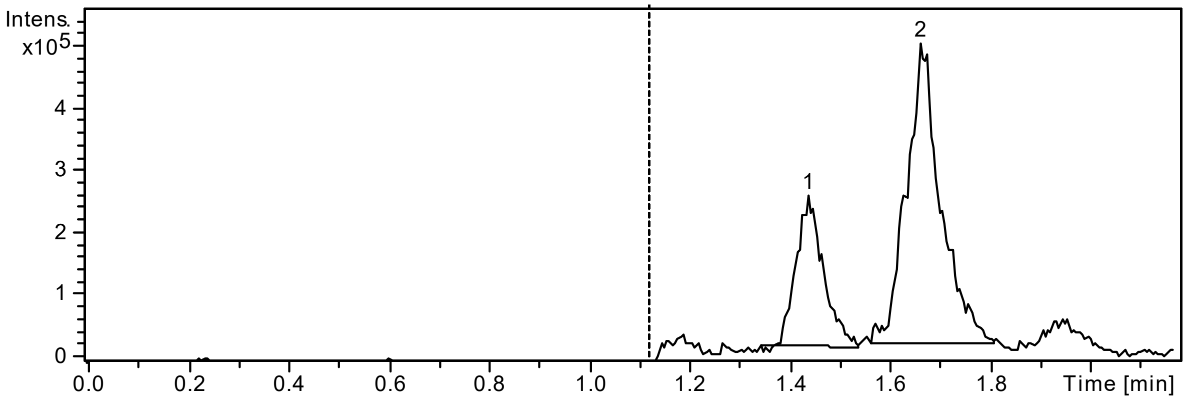

Figure S4. MS chromatogram of *Xanthium spinosum* L. S60 extract: (1) xanthatin, (2) 8-epi-xanthatin.
